# Ustiloxins and Ustilaginoidins in the Sclerotia Generated from Rice False Smut Balls and Their Contents

**DOI:** 10.3390/toxins18060264

**Published:** 2026-06-11

**Authors:** Dan Xu, Xuwen Hou, Liyao Liu, Xingyi Luo, Pengfei Wang, Jiankun Miao, Hai Dong, Daowan Lai, Ligang Zhou

**Affiliations:** 1 State Key Laboratory of Agricultural and Forestry Biosecurity, Department of Plant Pathology, College of Plant Protection, China Agricultural University, Beijing 100193, China; cauxudan@cau.edu.cn (D.X.); xwhou@cau.edu.cn (X.H.); lyliu@cau.edu.cn (L.L.); xingyiluo@cau.edu.cn (X.L.); pengfeiwang@cau.edu.cn (P.W.); dwlai@cau.edu.cn (D.L.); 2 Institute of Plant Protection, Liaoning Academy of Agricultural Sciences, Shenyang 110161, China; mjkkx@163.com (J.M.); lnsydh@163.com (H.D.)

**Keywords:** *Ustilaginoidea virens*, *Villosiclava virens*, rice false smut balls, sclerotia, ustiloxins, ustilaginoidins, mycotoxin distribution, mycotoxin content, gene expression analysis

## Abstract

Rice false smut (RFS) caused by the ascomycete *Ustilaginoidea virens* (teleomorph: *Villosiclava virens*) is a destructive fungal disease all over the world. The RFS balls are ball-like colonies transformed from individual grains through the infection of *U. virens*. The sclerotia, which are dormant structures, typically generate from the late-stage RFS balls, and produce ascospores that play a dominant role in the life cycle of the pathogen. *U. virens* can produce mycotoxins, mainly including ustiloxins and ustilaginoidins, that are toxic to rice plants and animals and pose a serious threat to their health. Though ustiloxins and ustilaginoidins have been isolated from the RFS balls, their distribution and contents in the sclerotia remain unclear. In this study, a systematic content analysis of main mycotoxins was conducted on the sclerotia and other parts of the late-stage RFS balls. Ustiloxins were predominantly found in the sclerotia and middle layer, whereas ustilagnoidins mainly accumulated in the outer and middle layers and rarely accumulated in the sclerotia and inner layer of RFS balls. The findings were further supported by transcriptome and RT–qPCR analysis data. The different accumulation and distribution of these two kinds of mycotoxins in the sclerotia and other parts of the RFS balls may be related to their specific physiological functions and deserve further investigation.

## 1. Introduction

**Figure 1:**
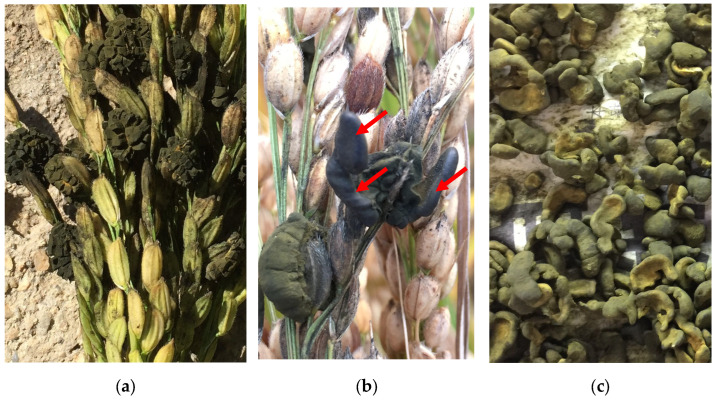
The sclerotia and RFS balls collected in Donggang, Liaoning Province of China on 14 October 2024. (**a**) The RFS balls without sclerotia; (**b**) The RFS balls containing sclerotia (as indicated by the arrows); (**c**) The collected sclerotia.

Rice false smut (RFS) is a destructive fungal disease caused by the pathogen *Ustilaginoidea virens* (teleomorph: *Villosiclava virens*) in paddy fields all over the world. The typical symptom is the formation of RFS balls in the spikelets. The black sclerotia are usually generated from the late-stage matured RFS balls due to significant diurnal temperature variation in the late autumn [1,2,3,4,5,6,7]. These sclerotia with hard structures consist of dense mycelia, and exhibit diverse shapes, such as horseshoe, irregular, oblong, or flattened shapes, with considerable size variation, typically measuring from 2 mm to 20 mm in length (Figure 1) [8,9]. In most cases, sclerotia are rarely found in the RFS balls. Sclerotia serve as the long-term survival dormant structures that persist in the soil or rice debris over 10 months, enabling them to overwinter. Upon encountering favorable conditions (e.g., adequate temperature and humidity) the following year, the fruiting bodies, including stromata, perithecia, asci, and ascospores, are developed from the sclerotia. The ascospores should be the primary inoculum source for new infections to rice plants. This suggests that the sclerotia play an important role in the life cycle of *U. virens* [10,11,12,13,14,15].

Ustiloxins and ustilaginoidins, two kinds of mycotoxins, have been previously isolated from the RFS balls. Ustiloxins belong to the cyclic peptides [16,17,18,19], and ustilaginoidins belong to bisnaphtho-γ-pyrone polyketides [20,21,22]. Both kinds of mycotoxins display diverse biological activities. Ustiloxins exhibit notably cytotoxic, antimitotic, and phytotoxic activities [16,17,23,24]. Ustiloxins are not only toxic to rice plants, but are also toxic to humans and animals [25,26,27,28,29]. Ustilaginoidins also show toxicity toward both animals and plants [21,22,30,31,32], along with antibacterial [21,33], and cytotoxic [21,22] activities. By contaminating rice food and feed, both ustiloxins and ustilaginoidins pose a serious threat to the health of humans and livestock.

A complete mature RFS ball was manually divided into five parts from the outer to the inner layer, namely sclerotia (S), outer layer (chlamydospores, C), middle layer (the complex of mycelia and immature chlamydospores, M), inner layer (the complex of mycelia and rice endosperm, R), and rice glume, respectively. The quantitative analysis revealed the content and distribution of main ustilaginoidins and ustiloxins in these layers of RFS balls at different maturation stages (i.e., early, middle and late stages). The glume portion was excluded due to the absence of detectable mycotoxins [34,35].

The content of ustilaginoidins was found to increase with RFS ball maturation, and was predominantly distributed in the outer and middle layers of RFS balls [34]. On the contrary, ustiloxins were primarily biosynthesized during the early stage of RFS ball development, and mainly accumulated in the mycelial layer [35]. However, the presence, abundance, and function of these mycotoxins in the sclerotia remain unclear.

In this study, we simultaneously quantified two kinds of major mycotoxins in the sclerotia and other parts of the late-stage RFS balls. Moreover, the comparative transcriptome and RT–qPCR analysis were further performed to verify the distribution and contents of ustiloxins and ustilaginoidins. This is the first time the content and distribution of mycotoxins in the sclerotia and RFS balls have been systematically analyzed. The findings are of great value for understanding the physiological and ecological roles of toxins in the survival, competition, and pathogenicity of *U. virens*.

## 2. Results

### 2.1. The Main Mycotoxins in the Sclerotia and Late-Stage RFS Balls

**Figure 2:**
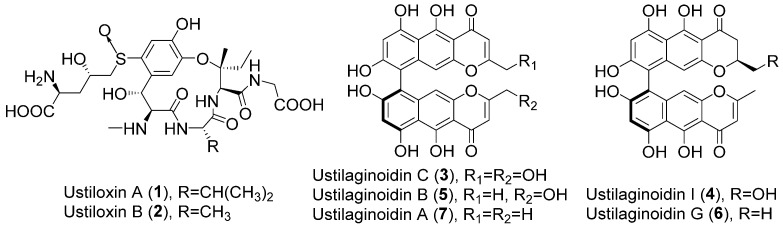
The structures of main ustiloxins and ustilaginoidins identified in the sclerotia of *U. virens*.

The sclerotia generated on the RFS balls are shown in Figure 1. Two major kinds of mycotoxins including ustiloxins (i.e., ustiloxins A and B) and ustilaginoidins (i.e., ustilaginoidins A, B, C, G and I) were identified and quantified in the sclerotia and other parts of the late-stage RFS balls with their structures shown in Figure 2.

#### 2.1.1. The Analysis of Ustiloxins

**Figure 3:**
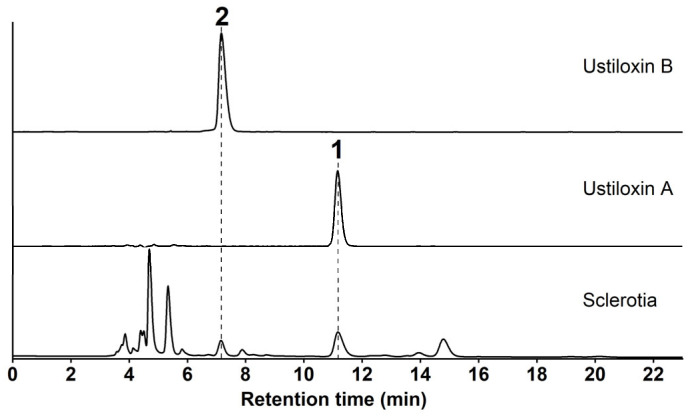
HPLC analysis of two main ustiloxins UA (**1**) and UB (**2**) and their corresponding authentic compounds in the sclerotia (λ = 220 nm).

Two main ustiloxins including ustiloxins A (UA, **1**) and B (UB, **2**) were detected in the late-stage RFS balls and their different parts including the outer layer, middle layer, inner layer, and sclerotia (Figure 3). The identification of UA (**1**) and UB (**2**) was accomplished by comparison with the authentic compounds and literature [35]. HPLC analysis showed that the HPLC profiles of all samples were similar to each other. The content of UA (**1**) was much higher than that of UB (**2**) in all samples, which showed the similar distribution trend. Due to the low contents of other ustiloxin analogs including ustiloxins G, C, D, and F in the samples, the combined content of UA and UB was estimated as the total ustiloxin content in the samples.

Quantitative analysis indicated that the contents of total ustiloxins (i.e., UA + UB) were comparable between the sclerotia (1346.54 µg/g) and middle mycelia layer (1486.93 µg/g) of RFS balls, which were significantly higher than those of the outer (chlamydospores) and inner (the complex of mycelia and rice endosperm) layers. The content of total ustiloxins (271.89 µg/g) in the inner layer was the lowest (Table 1).

#### 2.1.2. The Analysis of Ustilaginoidins

**Figure 4:**
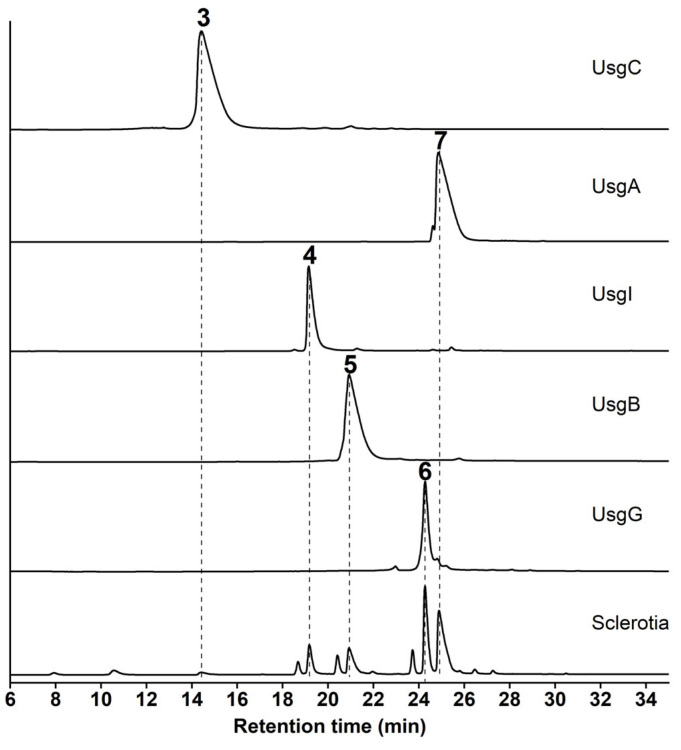
HPLC analysis of five main ustilaginoidins UsgC (**3**), UsgI (**4**), UsgB (**5**), UsgG (**6**) and UsgA (**7**) in the sclerotia and their corresponding authentic compounds.

HPLC analysis showed that ustilaginoidins C (**3**), I (**4**), B (**5**), G (**6**), and A (**7**) (abbr. as UsgC, UsgI, UsgB, UsgG, and UsgA, respectively) were the main ustilaginoidins in all samples (Figure 4), thereby the sum of these five compounds was roughly defined as the total ustilaginoidin amount produced in the samples. These compounds were identified by comparison with the authentic compounds and literature [34]. As shown in Table 2, the contents of total ustilaginoidins and their individual homologues in the samples were found to decrease in this order: outer layer > middle layer > sclerotia > inner layer. Specifically, the content in sclerotia was considerably lower than that in the outer or middle layer.

In the biosynthesis of ustilaginoidins, both 2(2′)-methyl hydroxylation and 2,3-double bond reduction were considered as post-modification [34,36]. According to whether the 2(2′)-methyl group of ustilaginoidins was hydroxylated, five main ustilaginoidins were divided into 2(2′)-hydroxylmethyl (i.e., UsgB, UsgC and UsgI) and 2(2′)-methyl (i.e., UsgA and UsgG) types. The proportions of 2(2′)-methyl hydroxylated ustilaginoidin amounts in the samples of the outer layer (47.61%) and middle layer (40.90%) over the total ustilaginoidin amounts were notably higher than those in the samples of sclerotia (29.76%) and inner layer (17.90%). In addition, the ratios of 2,3-dihydro-saturated ustilaginoidins (i.e., UsgG and UsgI), calculated as (G + I)/total, were significantly higher in the sclerotia (45.37%) and inner layer (42.69%) than those in the outer (17.01%) and middle (18.41%) layers.

The metabolic profiles of ustiloxins in the RFS balls and sclerotia were similar, but the differential profiles were observed for ustilaginoidins. In addition, the abundance of ustiloxins, including two main derivatives UA and UB in sclerotia, was much higher than that in other parts of the RFS balls, whereas the distribution of ustilaginoidins showed the opposite pattern. Due to the significant differences in chemical structures and toxic activities between these two types of mycotoxins (i.e., ustiloxins and ustilaginoidins), it is speculated that their distribution may be related to their specific physiological functions, and further research is warranted.

### 2.2. Transcriptome Results and Analysis

Transcriptome analysis was performed to compare the gene expression profiles among the whole RFS balls (Q) and their outer layer (C), middle layer (M), inner layer (R), and sclerotia (S). After sequencing quality control, a total of 15 samples yielded 303,760,571 clean reads and 90.56 Gb clean bases, with Q30 values greater than 94.74% (Table S1). 78.31–94.67% of reads were mapped to the *U. virens* reference genome (Table S1). These results indicated that the sequencing data were suitable for further analysis.

**Figure 5:**
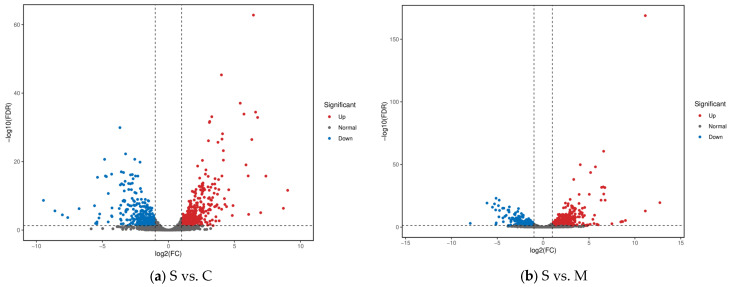

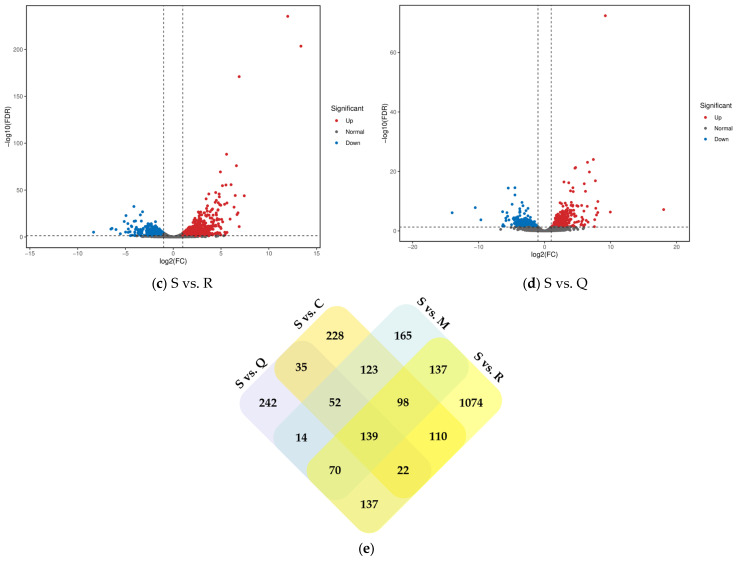
The differentially expressed genes (DEGs) in Q vs. S, C vs. S, M vs. S, and R vs. S. (**a**–**d**) Volcano plots of DEGs in C vs. S, M vs. S, R vs. S, and Q vs. S, respectively. (**e**) Venn diagram of DEGs. S, sclerotia; C, outer layer; M, middle layer; R, inner layer; Q, whole RFS ball.

The differentially expressed genes (DEGs) were identified using DESeq2 [37], with a false discovery rate (FDR) of <0.05 and a fold change of ≥2. Compared to the sclerotia portion (S), a total of 711, 807, 798, and 1787 DEGs were identified in the samples Q, C, M, and R, respectively. Among them, the numbers of upregulated genes were 388, 412, 452, and 1063, and the numbers of downregulated genes were 323, 395, 346, and 724, respectively (Figure 5a–d). Venn diagram analysis showed the common or specific DEGs among the different portions. Specifically, only 139 DEGs were found to be shared across all four pairwise comparisons (Figure 5e).

To further explore DEG functions, gene ontology (GO) and Kyoto Encyclopedia of Genes and Genomes (KEGG) pathway enrichment analyses were performed. Similar results were observed in four pairwise comparisons.

**Figure 6:**
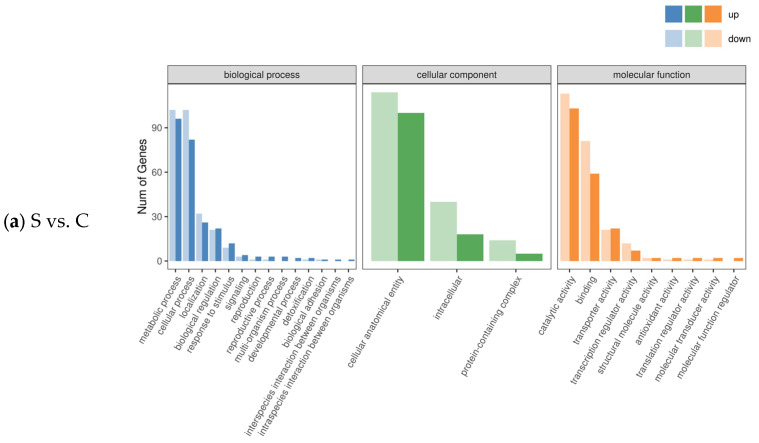

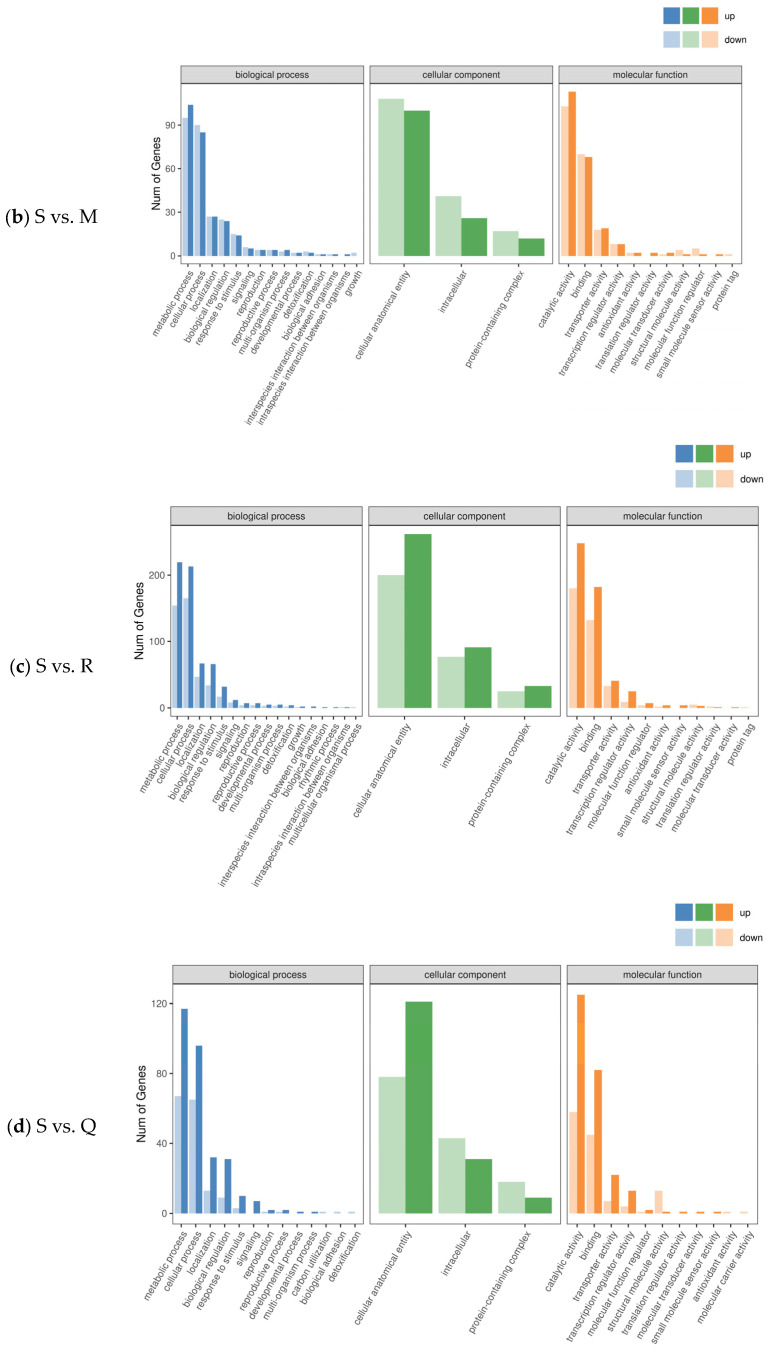
The most enriched gene ontology (GO) terms in (**a**) S vs. C, (**b**) S vs. M, (**c**) S vs. R and (**d**) S vs. Q. S, sclerotia; C, outer layer; M, middle layer; R, inner layer; Q, whole RFS balls.

The GO annotation of all unigenes grouped them into three main categories, including biological processes, cellular components, and molecular functions. Most enriched GO terms were mainly related to the metabolic process, cellular process, localization, biological regulation, response to stimulus, cellular anatomical entity, intracellular, protein-containing complex, catalytic activity, binding, transporter activity, and transcription regulator activity (Figure 6).

**Figure 7:**
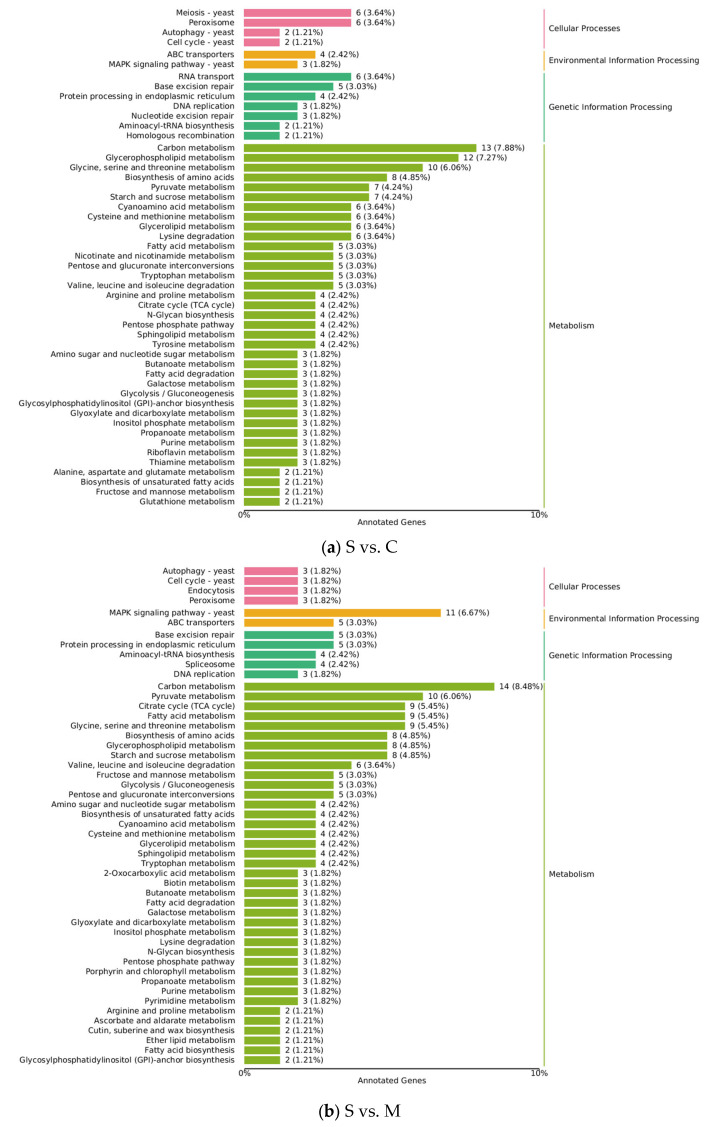

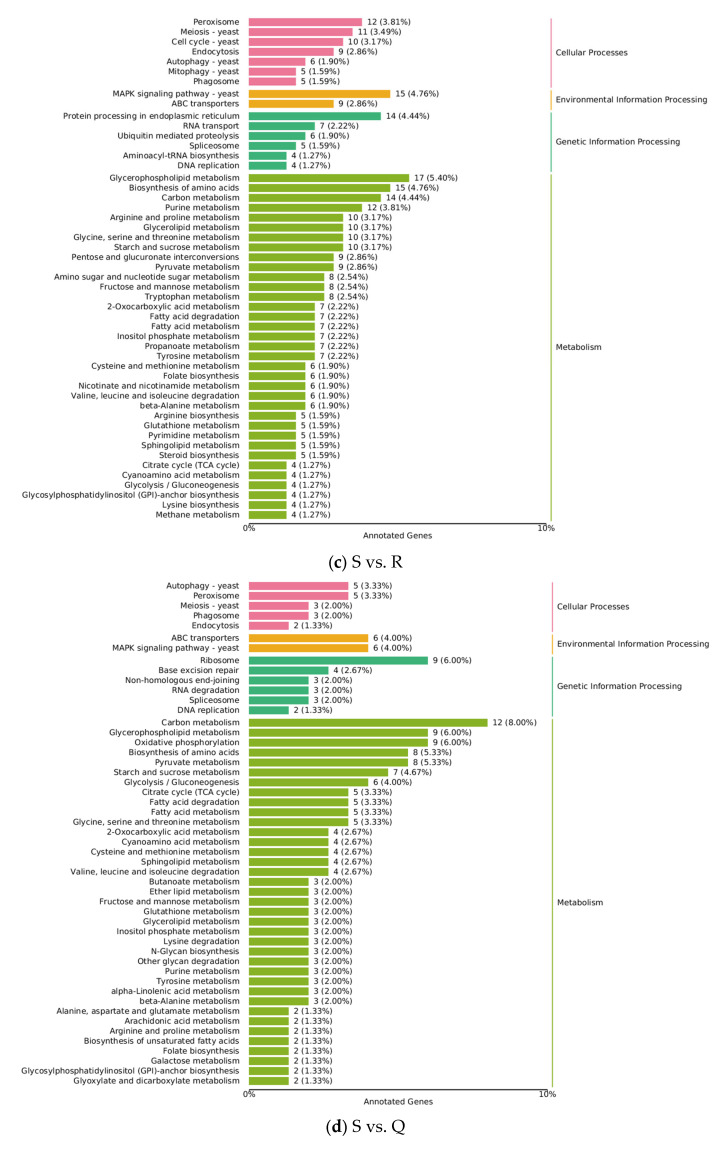
KEGG pathway enrichment classification. (**a**) S vs. C, (**b**) S vs. M, (**c**) S vs. R, and (**d**) S vs. Q. S, sclerotia; C, outer layer; M, middle layer; R, inner layer; Q, whole RFS balls.

**Figure 8:**
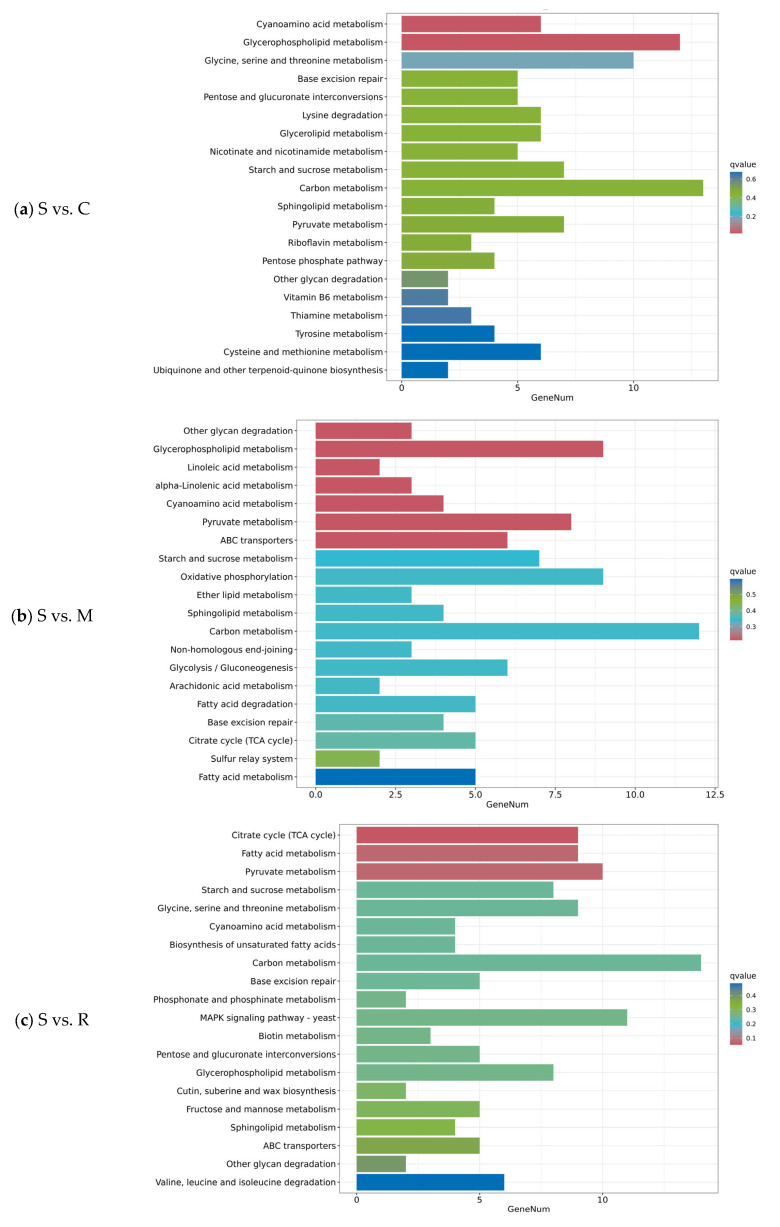

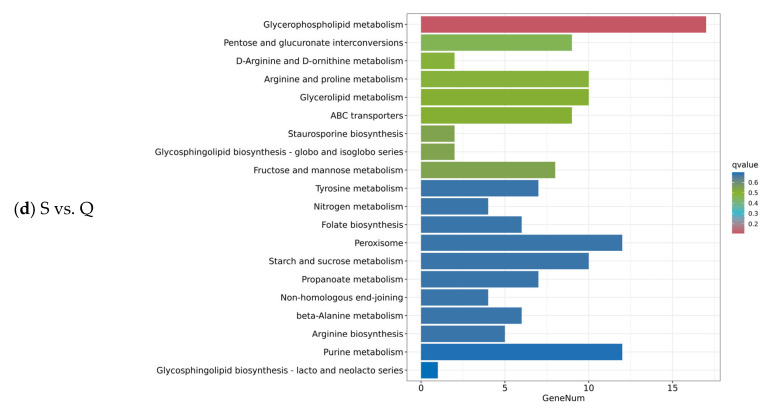
The top 20 enriched KEGG pathways in (**a**) S vs. C, (**b**) S vs. M, (**c**) S vs. R, and (**d**) S vs. Q. S, sclerotia; C, outer layer; M, middle layer; R, inner layer; Q, whole RFS ball.

KEGG analysis showed that the DEGs were successfully annotated to 121 pathways. As shown in Figure 7, fifty-one pathways were significantly enriched in S vs. C, S vs. M, S vs. R, and S vs. Q. They were categorized into four groups, including cellular processing, environmental information processing, genetic information processing, and metabolism. The top 20 enriched KEGG pathways were shown in Figure 8. Across the four pairwise comparisons, the main pathways included the glycerophospholipid metabolism, glycine metabolism, serine and threonine metabolism, carbon metabolism, oxidative phosphorylation metabolism, pyruvate metabolism, MAPK signaling pathway, purine metabolism, starch and sucrose metabolism, and peroxisome metabolism. It was noteworthy that the starch and sucrose metabolism, and glycerophospholipid metabolism were the most common among four comparisons. In addition, many metabolic pathways were closely associated with the vegetative growth, development, and pathogenicity of *U. virens* [38,39].

Although a large number of DEGs were found to exist among different parts of RFS balls, the differences between samples were likely not due to a single factor. Thus, we could not directly correlate these genes with differences in mycotoxin contents in RFS balls.

### 2.3. Analysis and Validation of Mycotoxin Biosynthetic Gene Expression

The biosynthetic gene clusters (BGCs) of ustiloxins and ustilaginoidins in *U. virens* have been reported previously [40,41,42,43,44,45,46]. The putative ustiloxin BGC (i.e., *UV8b_7484*–*UV8b_7494*) in *U. virens* was identified through homologous comparison with the corresponding BGC in *Aspergillus flavus* [40,47,48,49].

The BGC for ustilaginoidin biosynthesis has been identified in *U. virens*. Five structural genes (i.e., *UV8b_2086*, *UV8b_2087*, *UV8b_2089*, *UV8b_2090* and *UV8b_2091*) were demonstrated to participate in ustilaginoidin biosynthesis through gene knockout, *A. oryzae* heterologous expression, feeding experiments, and in vitro reactions [36,50,51,52].

**Figure 9:**
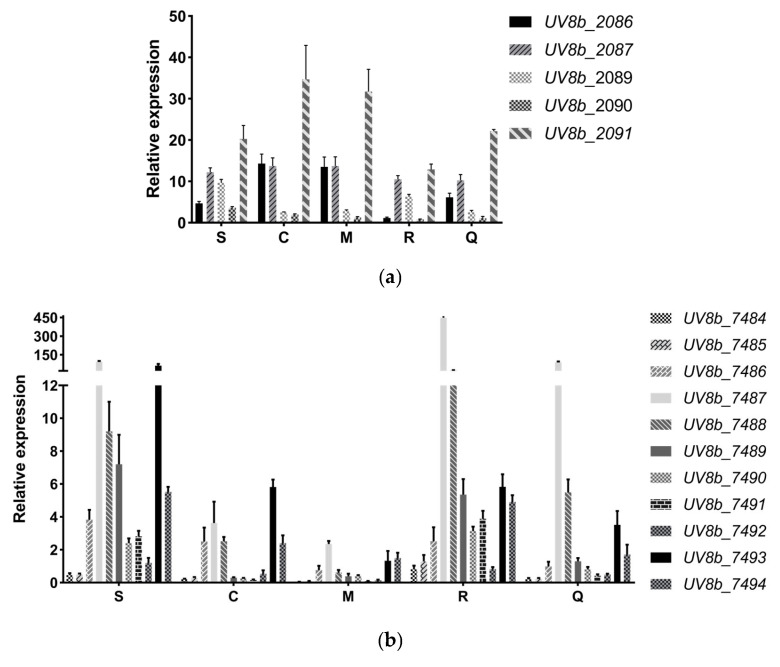
Relative expression of biosynthetic genes of two kinds of mycotoxins in sclerotia and other parts of RFS balls. (**a**) ustilaginoidins, and (**b**) ustiloxins. S, sclerotia; C, outer layer; M, middle layer; R, inner layer; Q, whole RFS ball.

Partial biosynthetic genes for ustilaginoidins were significantly upregulated in S vs. C, M, and Q, but downregulated in S vs. R, as observed in the transcriptome data (Table S2). To validate them, the expression levels of five identified biosynthetic genes of ustilaginoidins among the different samples were totally analyzed by RT-qPCR (Figure 9a). Obviously, the expression of the polyketide synthase gene *UV8b_2086* and laccase gene *UV8b_2091* followed the order of C > M > Q > S > R, which was consistent with the trend in ustilaginoidin content across different samples. No significant differences in expression were observed for the dehydratase gene *UV8b_2087* across all samples. The low expression of *UV8b_2090* may account for the trace amounts of 3-methyl ustilaginoidins in both RFS balls and sclerotia. In addition, the expression of *UV8b_2089* in S and R was higher than that in C, M, and Q, resulting in a decrease in the 2,3-dihydro-saturated ustilaginoidin ratio. Unfortunately, the gene responsible for 2-methyl hydroxylation has not yet been identified. However, given the consistent correlation between gene expression and the corresponding content observed for the other five genes, we speculated that the expression levels of this unknown gene in the parts C, M, and Q were higher than those in the parts R and S. For the four pairs of data (i.e., S vs. C; S vs. M; S vs. Q; and S vs. R), no common differentially expressed genes were selected.

The differential expression of ustiloxin biosynthetic genes was only observed in two pairwise comparisons (S vs. C and S vs. R). However, the downregulation of most ustiloxin biosynthetic genes in the sclerotia (compared to the inner part of RFS balls) was in contradiction with the higher ustiloxin content (Table S2). Further RT–qPCR revealed that the expression of biosynthetic genes in the sclerotia was much higher than in the RFS balls, consistent with the difference in toxin content between the two samples. However, the inner layer of RFS balls exhibited the highest expression level compared to the outer and middle layers, but the ustiloxin content in this part was the lowest (Figure 9b). The middle part showed the opposite results. The relatively high expression of biosynthetic genes was consistent with the high ustiloxin content detected in the sclerotia (Figure 9b).

## 3. Discussion

In this study, the distribution and content of ustilaginoidins and ustiloxins in sclerotia and other parts of late-stage matured RFS balls were systematically analyzed. Previous studies by our group revealed significant variations in the absolute contents of mycotoxins among the RFS balls from different geographical origins, while the distribution trends of toxins across different parts of the balls were highly similar [34,35]. For example, the total ustiloxin contents in the RFS balls from 14 different collection sites ranged from 332.2 µg/g to 1582.3 µg/g, showing no correlation with the collection sites, but the content of ustiloxin A was markedly higher than that of ustiloxin B in all samples [34]. Furthermore, the distribution pattern of different ustilaginoidins in the RFS balls shown in Table 2 in this study is consistent with that observed in the samples obtained from Sichuan Province of China [35].

As shown in Table 1, the contents of ustiloxins A and B were highest in sclerotia and the middle layer of RFS balls, and lowest in the inner part. It was speculated that ustiloxin biosynthesis mainly occurred in the sclerotia and the middle layer. However, RNA-seq and RT–qPCR data revealed that, except in sclerotia, the expression pattern of ustiloxin biosynthetic genes in different parts of the RFS balls was contrary to the corresponding toxin content. This is the first report on the correlation between ustiloxin contents and gene expression levels. The underlying reasons for this discrepancy remain unclear. We speculate that it may be related to the synthesis, transport, toxicity, or physiological/ecological functions of ustiloxins within fungal cells. In addition, the sclerotia serve as the dormant structures that can survive in soil or rice debris for more than ten months, enabling them to overwinter. Consequently, the substantial accumulation of highly toxic ustiloxins in sclerotia might be associated with specific biological functions, such as virulence-mediated primary infection or self-protection. These hypotheses require further experimental validation.

As shown in Table 2, the order of ustilaginoidin contents in different parts of the RFS balls was C > M > R, which was in line with the previous report [34]. Further analysis revealed that ustilaginoidin content in both the sclerotia and inner layer of the RFS balls was significantly lower than that in the outer and middle layers. Among them, the proportion of 2(2′)-hydroxylmethyl ustilaginoidins decreased, while the proportion of 2,3-dihydro-saturated ustilaginoidins increased. These results were further validated by transcriptome data and RT–qPCR analysis. The pigment compounds in fungal pathogens play key roles in protecting fungi against biotic [53] and abiotic stresses [54], and also serve as important virulence factors [55]. Ustilaginoidins, also belonging to pigment compounds in *U. virens*, may possess similar functions and require further investigation.

This study may provide some evidence for the elucidation of the physiological and ecological functions of ustilaginoidins and ustiloxins in *U. virens*. However, the sample collection from a single location and in a single year limited the generalizability of our conclusions. To further validate the results, more sclerotia samples from different regions and in various years will be collected to conduct a comprehensive analysis in the future.

## 4. Conclusions

Both the content and distribution of mycotoxins including ustiloxins and ustilaginoidins in the late-stage RFS balls containing sclerotia were systematically analyzed in this study. Compared with the outer and middle layers, both the sclerotia and inner layer exhibited a significantly lower ustilaginoidin content and a reduced proportion of hydroxylated derivatives, but a higher proportion of unsaturated ustilaginoidins. These findings were further confirmed by analyzing the expression levels of ustilaginoidin biosynthetic genes. In addition, the accumulation of ustiloxins in the sclerotia and middle layer was significantly higher than that in the outer and inner layers of RFS balls. However, the variation tendency of ustiloxin content across different parts of the RFS balls was inconsistent with their gene expression levels. The accumulation and distribution of mycotoxins may be related to their specific physiological functions in the RFS balls and sclerotia, which require further investigation.

## 5. Materials and Methods

### 5.1. Materials and Reagents

The late-stage RFS balls and sclerotia were collected on 14 October 2024 in Donggang (124.00° E, 40.12° N), Liaoning Province, China. The sclerotia were carefully separated from the RFS balls, and both the RFS balls and sclerotia were air-dried at room temperature (RT) before compound extraction. According to the previous reports [34,35], the RFS balls were also divided into five parts, including the sclerotia (S), outer layer (chlamydospores, C), middle layer (the complex of mycelia and immature chlamydospores, M), inner layer (the complex of mycelia and rice endosperm, R), and glume part. Due to the absence of detectable mycotoxins in the glume part at the late maturation stage, this tissue was omitted from subsequent analysis. The remaining four parts, including the sclerotia, outer layer, middle layer, and inner layer of the RFS balls, along with the whole RFS ball, were individually ground into a powder before extraction.

Reagents used in this study were obtained from Fisher Scientific (Waltham, MA, USA). Chromatographic grade methanol was applied for HPLC analysis, and analytical grade ethyl acetate (EtOAc) was employed for compound extraction. Ultrapure water was used for compound extraction and analysis.

### 5.2. HPLC Analysis of Ustiloxins and Ustilaginoidins

The quantitative analysis of mycotoxins was performed using a Shimadzu LC-20A HPLC system equipped with a SPD-M20A photodiode array detector (Shimadzu Corp., Tokyo, Japan), and a Phenomenex C18 column (i.d., 250 mm × 4.6 mm, 5 μm; Phenomenex Inc., Torrance, CA, USA).

#### 5.2.1. Analysis of Ustiloxins

A 100 mg aliquot of powdered sample from the different parts of RFS balls and the whole balls was weighed and extracted twice with 10 mL ultrapure water under ultrasonication at RT. The combined extracts were dried under a vacuum using a rotary evaporator. The residue was re-dissolved in 1 mL of ultrapure water and then filtered through a 0.22-μm millipore filter before HPLC analysis. Ustiloxins were quantified by HPLC with UV detection at 220 nm. The mobile phases were methanol (A) and H_2_O containing 0.02% TFA (B). The flow rate was 0.85 mL/min. The isocratic elution was applied using 15% A (*v*/*v*) for 25 min. The calibration curves for two ustiloxins (UA and UB) were established as previously described [35].

#### 5.2.2. Analysis of Ustilaginoidins

Ustilaginoidins from the different parts of RFS balls were extracted according to the previous report [34]. The sample weight was 10 mg. EtOAc was used for sample extraction and methanol was employed to re-dissolve dried residue prior to HPLC analysis. Ustilaginoidins were detected at 290 nm by HPLC. The mobile phases were methanol (A) and H_2_O containing 0.02% TFA (B). The flow rate was 0.85 mL/min. The gradient elution program was employed as follow: 0–5 min 50% A, 5–25 min 50–100% A, 25–35 min 100% A, 35–40 min 50% A. The ustilaginoidin content in the sclerotia was determined using intact specimens owing to their extreme hardness. The calibration curves for five main ustilaginoidins (i.e., UsgC, UsgA, UsgI, UsgB, and UsgG) were established according to the previous report [34].

### 5.3. Transcriptome Analysis

The samples, including the whole RFS balls (Q), and their outer layer (C), middle layer (M), inner layer (R), and sclerotia (S), were prepared and sent to BMKGENE Co., Ltd. (Beijing, China) for transcriptome analysis. Each sample had three replicates. The total RNA samples were extracted with Invitrogen TRIzol reagent. The concentration, purity, and integrity of the RNA were assessed using a NanoDrop 2000 spectrophotometer (Thermo Fisher Scientific, Wilmington, MA, USA) and the RNA Nano 6000 Assay Kit of the Agilent Bioanalyzer 2100 system (Agilent Technologies, Santa Clara, CA, USA). Then, mRNA-seq libraries were constructed using Hieff NGS Ultima Dual-mode mRNA Library Prep Kit for Illumina (Yeasen Biotechnology (Shanghai) Co., Ltd., Shanghai, China) and sequenced on an Illumina NovaSeq platform to generate 150 bp paired-end reads. The raw reads were further processed with the BMKCloud online platform (https://www.biocloud.net accessed on 23 February 2026).

### 5.4. RT-qPCR Analysis

The sclerotia-bearing RFS balls were collected from the field, and manually divided into five portions including sclerotia (S), outer layer (C), middle layer (M), inner layer (R), and rice glume. The total RNAs were isolated from the RFS balls and their four parts (i.e., S, C, M, and R) using the Trizol reagent (Invitrogen) and cDNAs were synthesized with the HiScript III 1st Strand cDNA Synthesis Kit (Vazyme, Beijing, China). The transcriptional levels of the biosynthetic genes of ustiloxins and ustilaginoidins were detected by RT-qPCR. The PCR was performed using SYBR Green qPCR Master Mix (Thermo Fisher Scientific, Waltham, MA, USA) with the α-tubulin gene as the internal reference. All primers were listed in Table S3. Three biological replicates were performed for each sample, and the reaction conditions were as follows: 95 °C for 3 min, followed by 40 cycles of 95 °C for 3 s, 56 °C for 30 s, and 72 °C for 10 s. Three technical replicates were included per biological replicate. The relative expression levels of genes were calculated by the 2^−ΔΔCT^ method [52,53,54].

### 5.5. Statistical Analysis

The experiments were conducted with ten replicates, and each datum was expressed as the mean value ± standard deviation (SD). All statistical analyses and charts were conducted using GraphPad Prism (version 8.0.2) software manufactured by GraphPad Software Inc. (San Diego, CA, USA). Significant differences were analyzed by one-way analysis of variance (ANOVA) using SAS version 9.4 (SAS Institute, Cary, NC, USA) and denoted by different letters at *p* ≤ 0.05.

## Figures and Tables

**Table 1:** The contents of ustiloxins A and B in the sclerotia and other parts of the RFS balls in the late maturation stage.

RFS Ball and Its Parts	Ustiloxin Content (μg/g)		
	UA (1)	UB (2)	UA + UB
Sclerotia (S)	1122.68 ± 244.02	223.86 ± 19.15	1346.54 ± 263.16 a*
Outer layer (C)	727.69 ± 74.73	129.58 ± 20.76	857.27 ± 91.36 b
Middle layer (M)	1209.61 ± 0.72	277.31 ± 57.41	1486.93 ± 56.69 a
Inner layer (R)	256.00 ± 34.92	23.83 ± 1.23	271.89 ± 29.18 d
Whole RFS ball (Q)	587.42 ± 25.65	66.75 ± 4.28	654.17 ± 24.79 c

* The values are expressed as means ± standard deviations. Different letters (i.e., a, b, c, and d) indicate significant differences among the samples in the same column at *p* ≤ 0.05 determined with SAS software.

**Table 2:** The contents of main ustilaginoidins in sclerotia and other parts of the RFS balls in late maturity stages.

RFS Ball and Its Parts	Ustilaginoidin Content (mg/g)						[(B + C + I)/ Total] (%)	[(G + I)/ Total](%)
	UsgC (3)	UsgI (4)	UsgB (5)	UsgG (6)	UsgA (7)	Total		
Sclerotia (S)	0.04 ± 0.02	0.32 ± 0.07	0.25 ± 0.08	0.61 ± 0.22	0.83 ± 0.27	2.05 ± 0.60 d*	29.76	45.37
Outer layer (C)	5.54 ± 1.35	8.12 ± 1.19	25.83 ± 3.33	5.99 ± 0.70	37.47 ± 4.14	82.94 ± 9.28 a	47.61	17.01
Middle layer (M)	2.81 ± 0.88	5.12 ± 1.04	16.17 ± 3.75	5.73 ± 1.36	29.10 ± 7.26	58.94 ± 13.05 b	40.90	18.41
Inner layer (R)	0.009 ± 0.005	0.05 ± 0.02	0.05 ± 0.03	0.21 ± 0.06	0.29 ± 0.09	0.60 ± 0.16 d	17.90	42.69
Whole RFS ball (Q)	2.47 ± 0.54	2.93 ± 0.74	8.98 ± 2.22	2.82 ± 0.77	12.55 ± 2.84	29.75 ± 6.28 c	48.34	19.33

* The values are expressed as means ± standard deviations. Different letters (i.e., a, b, c, and d) indicate significant differences among the samples in the same column at *p* ≤ 0.05 determined with SAS software.

## Data Availability

The original contributions presented in this study are included in the article/Supplementary Materials. Further inquiries can be directed to the corresponding author.

## Supplementary Materials

The following supporting information can be downloaded at: https://www.mdpi.com/article/10.3390/toxins18060264/s1, Table S1: The quality of RNA-seq data; Table S2: The mycotoxin-related DEGs among the whole RFS balls (Q) and their outer layer (C), middle layer (M), inner layer (R), and sclerotia (S); Table S3: The primers used for RT–qPCR.

## Abbreviations

The following abbreviations are used in this manuscript:

BGCs	biosynthetic gene clusters
DEGs	differential expression genes
EtOAc	ethyl acetate
GO	gene ontology
HPLC	high performance liquid chromatography
KEGG	Kyoto Encyclopedia of Genes and Genomes
PCR	polymerase chain reaction
RFS	rice false smut
RNA-seq	RNA-sequencing
RT	room temperature
RT-qPCR	reverse transcription quantitative PCR
SAS	statistical analysis system
UA	ustiloxin A
UB	ustiloxin B
UsgA	ustilaginoidin A (A)
UsgB	ustilaginoidin B (B)
UsgC	ustilaginoidin C (C)
UsgG	ustilaginoidin G (G)
UsgI	ustilaginoidin I (I)
vs.	Versus
UV	Ultraviolet
S	Sclerotia
C	outer layer (chlamydospores)
M	middle layer (the complex of mycelia and immature chlamydospores)
R	inner layer (the complex of mycelia and rice endosperm)
Q	whole RFS ball (balls)
