# Supplementary material for: Ustiloxins and Ustilaginoidins in the Sclerotia Generated from Rice False Smut Balls and Their Contents
Source: Toxins (Basel). 2026 Jun 11;18(6):264. doi: 10.3390/toxins18060264 (PMC13307881; doi:10.3390/toxins18060264)
Supplement: Supplementary file 1 [file toxins-18-00264-s001.zip › toxins-4195884-supplementary.pdf]

Supplementary Materials

Ustiloxins and Ustilaginoidins in the Sclerotia Generated from Rice False Smut Balls and Their Contents

Table S1. The quality of RNA-seq data.

Sample	Clean reads	Clean bases (G)	Q20 (%)	Q30 (%)	GC Content (%)	Mapping Efficiency (%)
C_1	20677744	6.18	98.11	95.05	56.85	88.84
C_2	19978817	5.96	98.01	94.83	57.45	91.30
C_3	20336792	6.08	98.12	95.00	57.03	90.68
M_1	19166593	5.72	97.99	94.74	56.90	91.13
M_2	20680211	6.18	98.09	95.07	56.64	90.59
M_3	19686992	5.88	98.01	94.80	56.81	78.31
R_1	20010298	5.95	99.38	97.80	56.25	94.67
R_2	20032198	5.99	97.97	94.77	56.87	94.27
R_3	21022778	6.28	98.01	94.92	56.77	92.43
B_1	20294632	6.00	98.06	95.07	55.77	90.58
B_2	22110105	6.53	98.54	96.20	55.64	83.33
B_3	19485939	5.78	98.29	95.52	56.45	94.34
S_1	19493115	5.83	98.27	95.46	57.34	94.23
S_2	20292818	6.07	98.14	95.15	57.48	92.03
S_3	20491539	6.12	98.10	94.96	57.15	91.40
Total	303760571	90.56				

Table S2. The mycotoxin-related DEGs among the whole RFS balls (Q) and their outer layer (C), middle layer (M), inner layer (R), and sclerotia (S).

S vs. C/M/Q/R	Mycotoxin	Gene	FPKM						FDR	Log2FC	Down/Up
S vs. C			C1_FPKM	C2_FPKM	C3_FPKM	S1_FPKM	S2_FPKM	S3_FPKM			
	Ustilaginoidins	gene-UV8b_2087	1632.342651	2511.41626	2214.497314	958.70636	868.67218	498.866882	0.00292961359405063	-1.3313	Down
		gene-UV8b_2088	130.917862	204.749985	171.662186	83.653259	67.101242	41.304405	0.00549405560977754	-1.2870	Down
	Ustiloxins	gene-UV8b_7485	0.635019	1.866441	1.495584	6.340772	6.161984	8.352048	0.000273210247876371	2.49593782941077	Up
		gene-UV8b_7486	2.701779	5.976433	4.470524	15.014652	12.419276	24.914473	0.000309413304555105	2.11119503900425	Up
		gene-UV8b_7488	26.83872	59.526905	29.105497	81.231026	67.329323	148.138916	0.0425555312856224	1.46819526544765	Up
		gene-UV8b_7489	9.622587	18.929258	14.333042	32.202362	35.347172	63.468742	0.00572563347226731	1.73602738345099	Up
S vs. M			M1_FPKM	M2_FPKM	M3_FPKM	S1_FPKM	S2_FPKM	S3_FPKM			
	Ustilaginoidins	gene-UV8b_2087	2732.297119	3970.061279	1257.456177	958.70636	868.67218	498.866882	0.005222005786043	-1.7182	Down
		gene-UV8b_2088	211.699539	239.462036	89.368217	83.653259	67.101242	41.304405	0.0238808610338211	-1.4280	Down
		gene-UV8b_2091	6185.625977	7732.89502	3033.061035	2024.697754	2213.179199	1087.708618	0.00619332996367433	-1.5979	Down
S vs. Q			Q1_FPKM	Q2_FPKM	Q3_FPKM	S1_FPKM	S2_FPKM	S3_FPKM			
	Ustilaginoidins	gene-UV8b_2088	270.719391	304.07251	159.63826	83.653259	67.101242	41.304405	0.00765818884901149	-1.9409	Down

Table S2. *Cont.*

S vs. C/M/Q/R	Mycotoxin	Gene	FPKM						FDR	Log2FC	Down/Up
			R1_FPKM	R2_FPKM	R3_FPKM	S1_FPKM	S2_FPKM	S3_FPKM			
S vs. R	Ustilagninoidins	gene-UV8b_2086	33.610703	48.615891	248.282425	1092.234497	1160.095825	359.683105	0.00573467406424476	3.2138	Up
		gene-UV8b_2087	142.068817	207.378204	604.003662	958.70636	868.67218	498.866882	0.00999719310582109	1.4992133194208	Up
	Ustiloxins	gene-UV8b_7484	3646.285156	1200.375732	456.180298	13.652365	12.651259	31.066946	7.14415222217274e-10	-6.4065	Down
		gene-UV8b_7485	1066.428711	277.684082	85.988869	6.340772	6.161984	8.352048	1.21771934997657e-08	-5.9887	Down
		gene-UV8b_7486	489.013	175.90274	54.061153	15.014652	12.419276	24.914473	2.19951822669502e-07	-3.6625	Down
		gene-UV8b_7487	129906.125	32941.16797	16754.34766	314.774109	524.331726	946.449097	3.72870496667918e-09	-6.5222	Down
		gene-UV8b_7488	7358.574707	2582.292725	1369.813599	81.231026	67.329323	148.138916	3.00266735939145e-17	-5.1265	Down
		gene-UV8b_7489	2525.275879	826.294556	495.8927	32.202362	35.347172	63.468742	5.37794958054416e-15	-4.7451	Down
		gene-UV8b_7490	1295.191284	451.263794	308.840973	17.288342	21.982265	50.055557	1.99808650144955e-12	-4.3813	Down
		gene-UV8b_7491	2119.903809	554.469849	295.352295	22.100344	31.926264	50.015152	1.13799381134912e-05	-4.7077	Down
		gene-UV8b_7492	237.153122	88.81189	55.459503	6.644325	5.817981	11.576864	5.06076280641300e-11	-3.8554	Down
		gene-UV8b_7493	1397.981445	337.956146	153.658371	27.351339	16.845936	11.956056	5.93082426879103e-06	-4.9851	Down
		gene-UV8b_7494	391.915009	453.049347	478.910126	148.542938	89.474594	105.405128	4.01218133210874e-11	-1.7832	Down

Table S3. The primers used for RT–qPCR.

Primer Name	Sequence (5′→3′)
qRT-Uv8b-7484-F	accccatgccattagtccct
qRT-Uv8b-7484-R	aggacagcgagactttggag
qRT-Uv8b-7485-F	cgctctcgctcttgaagttgg
qRT-Uv8b-7485-R	atatgatgatgggcctctcgc
qRT-Uv8b-7486-F	agtcagtctccctccgcaac
qRT-Uv8b-7486-R	ggatctcggtgtggatgtcg
qRT-Uv8b-7487-F	gctcatctccatcttggccac
qRT-Uv8b-7487-R	atggcgtagtcctcaacagagg
qRT-Uv8b-7488-F	gatgagcggcctaaatcaggg
qRT-Uv8b-7488-R	gcacgaatgcaacaagaaggg
qRT-Uv8b-7489-F	tcaacgcccatttcgacact
qRT-Uv8b-7489-R	ggacggtgatttgctctgcc
qRT-Uv8b-7490-F	acacggtcagtcaacatcctt
qRT-Uv8b-7490-R	tcaaggtgtaacgatgccagg
qRT-Uv8b-7491-F	ccaaggccatgctacgataatct
qRT-Uv8b-7491-R	ccaatccgttcggatgtctcttg
qRT-Uv8b-7492-F	accgtaatctcaagaccaacgtt
qRT-Uv8b-7492-R	gaggtattgctggacaaggtgg
qRT-Uv8b-7493-F	aggcaatgacaagcgcatg
qRT-Uv8b-7493-R	ttcagcgactcgaggctca
qRT-Uv8b-7494-F	gtatctcccaagggcttgct
qRT-Uv8b-7494-R	gatgtggctgatggtttcgc
qRT-Uv8b-2086-F	cagacaagtcgctcatcctc
qRT-Uv8b-2086-R	tgaagaagcccgagcacttt
qRT-Uv8b-2087-F	aggcatctcgaccgaagact
qRT-Uv8b-2087-R	atctgcacgcagcaatcgta
qRT-Uv8b-2088-F	ctgtcggggaccaggaagaa
qRT-Uv8b-2088-R	gagatggccaccatgatgac
qRT-Uv8b-2089-F	cggagtgtgtttgcgagatt
qRT-Uv8b-2089-R	tgaagaacatggcaatcgacat
qRT-Uv8b-2090-F	gtgtacacggacatctcagc
qRT-Uv8b-2090-R	tgcaattcgtcgacacaacc
qRT-Uv8b-2091-F	cggccacggagaagtatact
qRT-Uv8b-2091-R	cagactgtatatgggctggg
qRT-tublin-F	ggcgtttacaatggcacttc
qRT-tublin-R	cggaacagttgaccaaaagg
